# Supplementary material for: Machine learning for quantum dynamics: deep learning of excitation energy transfer properties
Source: Chem Sci. 2017 Oct 23;8(12):8419–26. doi: 10.1039/c7sc03542j (PMC5863613; doi:10.1039/c7sc03542j)
Supplement: Supplementary file 1 [file SC-008-C7SC03542J-s001.pdf]

Electronic Supplementary Material (ESI) for Chemical Science.
This journal is © The Royal Society of Chemistry 2017

Machine Learning for Quantum Dynamics: Deep Learning of Excitation Energy Transfer Properties

Florian Häse, Christoph Kreisbeck,* and Alán Aspuru-Guzik†

Department of Chemistry and Chemical Biology, Harvard University, Cambridge, Massachusetts, 02138, USA
(Dated: October 19, 2017)

SUPPLEMENTARY INFORMATION

A. Modeling of excitation energy transfer

The energy transport in light-harvesting complexes is determined by coupled pigments which are embedded in a protein scaffold.[1,2] The large number of degrees of freedom in the system renders *ab initio* calculations on the atomistic level impossible. Therefore, the exciton transfer dynamics is typically modeled with an effective Frenkel exciton Hamiltonian.[3,4] The exciton Hamiltonian for a system of N sites for the single exciton manifold reads

$$H_{\text{system}} = \sum_{i=1}^{N} \varepsilon_i |i\rangle\langle i| + \sum_{i\neq j}^{N} V_{ij} |i\rangle\langle j|, \tag{1}$$

where ε_i denotes the first excited state energy of the i-th pigment molecule and V_{ij} denotes the Coulomb coupling between excited states at the i-th and j-th molecule. We assume that the exciton system couples linearly to the vibrational environment of each pigment, which is assumed to be given by a set of harmonic oscillators. The phonon mode dependent interaction strength is captured by the spectral density

$$J_i(\omega) = \pi \sum_k \hbar^2 \omega_{i,k}^2 d_{i,k}^2 \delta(\omega - \omega_{i,k}). \tag{2}$$

Here, $d_{i,k}$ defines the coupling of the k-th phonon mode $(b_{i,k}^\dagger)$ of the i-th pigment with frequency $\hbar\omega_{i,k}$.

In the first step of photosynthesis, energy is absorbed in the antenna pigments and subsequently transferred to the reaction center in which photochemical reactions are triggered. Within a simple picture, this process can be modeled by energy transfer from an initially excited pigment (donor) to a target state (acceptor). We model energy trapping in the acceptor state $|\text{acceptor}\rangle$ phenomenologically by introducing anti-Hermitian parts in the Hamiltonian

$$\mathcal{H}_{\text{trap}} = -i\hbar\Gamma_{\text{trap}}/2\, |\text{acceptor}\rangle\langle\text{acceptor}|, \tag{3}$$

where Γ_{trap} defines the trapping rate. In a similar way, we model radiative or non-radiative decay to the electronic ground state as exciton losses

$$\mathcal{H}_{\text{loss}} = -i\hbar\Gamma_{\text{loss}}/2 \sum_i |i\rangle\langle i|. \tag{4}$$

The rate $\Gamma_{\text{loss}}^{-1}$ defines the exciton lifetime. In this study we are interested in two different exciton propagation characteristics: the average transfer time

$$\langle t\rangle = \Gamma_{\text{trap}}/\eta \int_0^{t_{\max}} \mathrm{d}t\, t\, \langle\text{acceptor}|\rho(t)|\text{acceptor}\rangle, \tag{5}$$

---

* christophkreisbeck@gmail.com
† aspuru@chemistry.harvard.edu

and the overall efficiency

$$\eta = \int_0^{t_{max}} \mathrm{d}t\, \Gamma_{\mathrm{trap}} \langle \mathrm{acceptor}|\rho(t)|\mathrm{acceptor}\rangle, \tag{6}$$

which corresponds to the accumulated trapped population during the transfer process. For numerical evaluations, we replace the upper integration limit by t_{max} which is chosen such that the total population within the pigments has dropped below 0.0001.

The exciton dynamics is expressed in terms of the reduced density matrix $\rho(t)$, which can be obtained from standard open quantum system approaches. Here we employ the hierarchical equations of motion (HEOM) approach which accounts for the reorganization process,[5–8] in which the vibrational coordinates rearrange to their new equilibrium position upon electronic transition from the ground to the excited potential energy surface. The major drawback of the HEOM approach is the adverse computational scaling, which arises from the need to propagate a complete hierarchy of auxiliary matrices. Therefore, we employ a high-performance implementation of HEOM integrated into the *QMaster* software package.[9–11] As demonstrated in previous publications, *QMaster* enables HEOM simulations for large systems comprising of up to hundred pigments,[12] as well as to perform accurate calculations for highly structured spectral densities.[10,11,13,14]

A computationally much cheaper formalism, the Redfield approach, can be derived with the assumption of weak couplings between the system and the bath in combination with a Markov approximation.[4,15] The secular approximation simplifies the equation even further and allows to write the dynamics in the form of a Lindblad master equation. This drastically reduces the computational demand of this approach compared to exciton propagation under the HEOM, which explains the popularity of the secular Redfield equations. However, secular Redfield has been shown to underestimate the transfer times in certain light-harvesting complexes.[16]

B. Exciton transfer properties of biological Hamiltonians

We report the exciton transfer times calculated for the four light-harvesting complexes (LHCs) FMO, RC, CP43+RC and CP47+RC.[17–20] Exciton transfer times were obtained from HEOM calculations, secular Redfield calculations and MLP predictions. Results are listed in Tab. I. For all four biological complexes, we find that the MLP predictions are more accurate than the secular Redfield calculations.

LHC	Exciton transfer time [ps]			Computation time		
	HEOM	MLP	Secular Redfield	HEOM (GPU)	MLP (CPU)	Secular Redfield (CPU)
FMO	5.78	4.99	4.18	2.1 min	5 ms	14.5 min
RC	7.94	7.52	7.48	3.9 min	3 ms	8.9 min
CP43+RC	13.80	11.64	11.16	7.4 h	5 ms	14.6 h
CP47+RC	18.92	19.31	15.08	31.2 h	4 ms	40.9 h

TABLE I. Comparison of exciton transfer times for the light-harvesting complexes (LHCs) considered in this study computed with the hierarchical equations of motion (HEOM), multi-layer perceptrons (MLPs) and secular Redfield. We report the obtained exciton transfer times as well as the runtimes of the calculations. Note, that HEOM calculations were run on GPUs, while MLP predictions and secular Redfield calculations were run on CPUs. MLPs were trained on PCA selected training sets and neither of the LHCs was included in the training sets

C. Distributions of sampled Frenkel exciton Hamiltonians

In this study we generated four datasets to train MLPs for excitation energy transport property predictions. For each dataset we randomly sampled excited state energies and inter-site couplings of Frenkel exciton Hamiltonians from uniform distributions within particular parameter value ranges inspired by existent light-harvesting complexes (see main text for details).

The four generated datasets differ in both, the parameter value ranges and the number of excitonic sites and thus the number of parameters in the Frenkel exciton Hamiltonians. MLPs are trained on 12000 Frenkel exciton Hamiltonians sampled from each of the four spanned parameter spaces. Since MLP models have only limited extrapolation

capabilities, the training set for MLP models needs to sufficiently cover the parameter space of interest such that test set points are close to training set points. As MLP models are fully connected, even a deviation in only one of the input parameters can cause significantly different predictions.

We estimate the relative coverage of the parameter spaces of the four investigated datasets by computing the distance between all Hamiltonians in each dataset based on the maximums norm as defined in Eq. 7

$$\Delta_{\max}(A,B) = \max_{i,j=1,\ldots,n} |a_{ij} - b_{ij}|, \tag{7}$$

where both A and B are symmetric matrices with $A, B \in \mathbb{R}^{n\times n}$. Fig. 1 illustrates the obtained distributions.

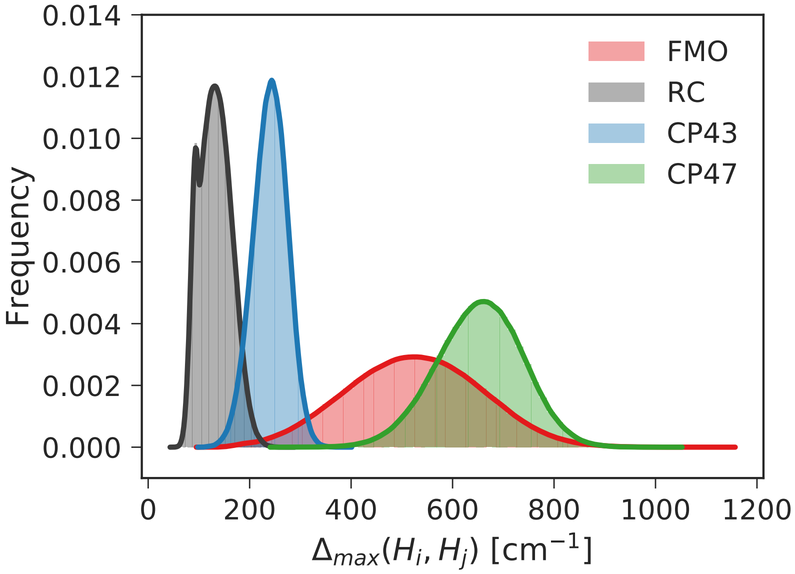

FIG. 1. Distributions of distances between Frenkel exciton Hamiltonians evaluted with the maximums norm for each of the four datasets.

We observe much larger distances between Hamiltonians in the FMO and CP47 datasets compared to the RC and CP43 datasets. Based on this observation, we expect lower prediction accuracies of trained MLP models for the FMO and the CP47 dataset which is consistent with the obtained results in Tab. 2 in the main text.

D. Computational cost of exciton dynamics calculations

Established methods for computing the population dynamics of excitonic systems such as the hierarchical equations of motion (HEOM) approach suffer from adverse computational scaling. Because of this drawback less sophisticated techniques with lower computational demand such as the secular Redfield method are more popular. Both methods need to run a full population dynamics calculation for obtaining exciton transfer properties such as the average transfer time or transfer efficiency.

We report the runtimes of HEOM and secular Redfield calculations observed during the generation of the four datasets used in this study. Each dataset consists of 12000 randomly generated exciton Hamiltonians for which we computed average transfer times and transfer efficiencies with both methods (see main text for details). HEOM calculations were carried out in the high-performance *QMaster* package,[9–11] which uses the architecture of GPUs for propagating a complete hierarchy of auxiliary matrices in parallel. Secular Redfield calculations were run on single core CPUs. Computation time for the individual Hamiltonians show some variations in computation time since depending on the excitation energy transfer times, we need to run the exciton dynamics for a larger or smaller number of time-steps. In Tab. II we show the average computation time for a single exciton Hamiltonian for each dataset (average over all 12,000 Hamiltonians), as well as the total computational cost to generate the complete datasets.

As a compromise between accuracy and computational costs we truncate the hierarchy at $N_{\mathrm{max}} = 5$ for the datasets with fewer pigments (RC and FMO) and at $N_{\mathrm{max}} = 4$ for the datasets with more pigments (CP43 and CP47). We tested that the accuracy is retained at these truncation levels by simulating a random selection of 10 exciton Hamiltonians drawn from each dataset at respective lower and higher levels of truncation. We observe an average 1.6 % deviation in the calculated transfer times between the higher and lower truncation levels for the FMO dataset. The RC and CP43 datasets show smaller deviations with 1.1 % for RC and 0.1 % for CP43. For the CP47 we observe a deviation of about 2.4 %. Despite the small deviations induced by the earlier truncation of the hierarchy, we use the lower truncation level in our dataset generation to keep the computational costs at a reasonable level (see Tab. II).

While transfer properties in smaller systems, as they are modeled in the RC and FMO datasets, can be calculated within a few minutes, calculations on larger systems modeled in the CP43 and CP47 datasets show significantly higher computational demands with typical runtimes in the order of 6 to 18 hours. The significant computation times illustrate that large-scale simulations on artificial exciton systems can be computationally quite exhaustive.

Multi-layer perceptrons (MLPs), however, encode a set of matrix operations, which allows for a significantly faster calculation of the properties of interest. By construction, the time for computing the output of an MLP scales linearly with the number of neurons in the architecture. We find that our trained MLP models could predict exciton transfer times and transfer efficiencies of one complete dataset introduced in this study in less than 10 seconds on a single CPU. This estimate includes the time spent for loading all 12000 exciton Hamiltonian matrices into memory and running the matrix operations encoded in the MLP architecture as well as writing the results of the prediction to file.

Dataset	HEOM (GPU)		MLPs (CPU)		Secular Redfield (CPU)	
	T_{single}	T_{all}	T_{single}	T_{all}	T_{single}	T_{all}
FMO	4.0 min	33.6 days	<0.1 ms	0.3 s	8.8 min	73.5 days
RC	2.2 min	18.3 days	0.1 ms	1.6 s	4.4 min	36.3 days
CP43	5.8 h	7.9 years	0.6 ms	7.5 s	10.7 h	14.7 years
CP47	18.2 h	24.9 years	0.1 ms	1.5	36.6 h	41.9 years

TABLE II. Runtimes T for exciton transfer time computations carried out with the *QMaster* package, version 0.2,[9–11] for all four datasets. We report runtimes for the entire datasets, T_{all}, each comprising of 12000 exciton Hamiltonians, as well as the average runtime per exciton Hamiltonian, T_{single}. Hierarchical equations of motion (HEOM) calculations were carried out on a NVIDIA Tesla K80 GPU and secular Redfield calculations as well as multi-layer perceptron (MLP) predictions on Intel(R) Xeon(R) CPUs X5650 @ 2.67 GHz.

MLP models can be considered a valid alternative to secular Redfield calculations when running exciton dynamics calculations on a large number of excitonic systems due to the comparable accuracy at an orders of magnitude lower computational cost.

E. Dataset preparations for multi-layer perceptron predictions

MLP models constructed in this study were designed to predict exciton transfer times and transfer efficiencies from the Frenkel exciton Hamiltonian of an excitonic system. The numerical values of excited state energies and inter-site couplings in the exciton Hamiltonian, as well as, the transfer times and transfer efficiencies depend on the chosen unit system. Further, the ranges of these properties can be very different.

Features and targets with significantly different numerical values are more challenging to learn for most machine learning models as the applied model first has to learn the general range of numerical values before it can learn more subtle differences. The training procedure of machine learning models in general and MLPs, in particular, can be accelerated by rescaling features and targets to similar numerical values.

Instead of providing the Frenkel exciton Hamiltonians as training features and the transfer times and efficiencies as training targets in a particular system of physical units we rescaled both features and targets based on the parameter ranges in the training set to facilitate faster and more stable MLP training. In particular, we subtracted the training set mean from all excited state energies in the exciton Hamiltonians and then mapped all feature elements h_{ij} onto the interval $[-2, 2]$ (see Eq. 8), where h_{ij} denotes a particular element of a particular exciton Hamiltonian and H_{train}

denotes the set of all exciton Hamiltonians in the training set. The rescaled features are denoted with $\tilde{h}_{ij}$. Rescaling the input features onto the $[-2, 2]$ interval ensures that all input values lie within sensitive regions of input layer activation functions, which avoids neuron saturation at the beginning of the MLP training.

$$\tilde{h}_{ij} = 4\frac{h_{ij} - h_{\min}}{h_{\max} - h_{\min}} - 2, \qquad h_{\min} = \min_{h_{ij} \in H_{\text{train}}} (h_{ij}), \qquad h_{\max} = \max_{h_{ij} \in H_{\text{train}}} (h_{ij}). \tag{8}$$

Prediction targets need to be rescaled onto an interval, which lies within the codomain of the output layer activation function. The target properties in this study are exciton transfer times and efficiencies. Both of these properties are always positive, but while efficiencies also have an upper bound, transfer times could a priori be arbitrarily large. We, therefore, chose the softplus function for the activation function of the MLP output layer as it exhibits similar properties. To use most of the non-linear regime of the softplus function, we decided to map our training targets t (the collective set of transfer times and efficiencies) onto the interval $(0, 4]$ based on the maximum transfer time and efficiency in the training set T_{train} (see Eq. 9). Exciton transfer times and transfer efficiencies were rescaled separately. Note, that both exciton transfer times and efficiencies are always positive which justifies the implicit lower bound of zero in the equation.

$$\tilde{t} = \frac{4t}{t_{\max}}, \qquad t_{\max} = \max_{t \in T_{\text{train}}} (t) \tag{9}$$

F. Bayesian optimization for hyperparameter selection

Multi-layer perceptrons (MLP) consist of a set of neurons organized in layers. Each neuron accepts an input, which is rescaled by a set of weights and biases intrinsic to the neuron, to calculate its output. The outputs of neurons in one layer are propagated through the MLP as the input for the subsequent layer. While weights and biases of each neuron are collectively referred to as the parameters of the MLP, an MLP model contains additional free parameters such as the number of layers and the number of neurons per layer. The latter are the hyperparameters of the model. MLP parameters are typically optimized on the training set with gradient based optimization techniques such as stochastic gradient descent. Hyperparameters are instead selected by optimizing the parameters of an MLP on the training set and evaluating the prediction accuracy on the validation set.

In this study, we decided to employ a Bayesian optimization approach to find hyperparameters for accurate MLP architectures. We chose a total of six hyperparameters to be optimized and set fixed ranges for each of them for the Bayesian optimization. Hyperparameters and ranges are reported in Tab. III. In particular, we also included the number of training points as a hyperparameter to investigate by how much the prediction accuracy of MLPs can increase when expanding the training set.

Hyperparameter	low	high
Training points	4000	6000
Network layers	3	18
Neurons per layer	2	2000
Learning rate	10^{-7}	10^{-1}
Regularization	10^{-18}	10^{6}
Activation function	sigmoid, relu, tanh softsign, softplus	

TABLE III. Selection of hyperparameters for Bayesian optimization of the MLP architecture. Lower and upper bounds were applied to the search space for five of the six parameters. Five different options were provided to the Bayesian optimizer for choosing an activation function for all but the last network layer

The particular ranges for individual hyperparameters were chosen based on a few test training runs and chosen large enough that the Bayesian Optimizer is able to explore diverse MLP architectures. Although we found that MLPs perform more accurately when using more points in the training set, we restricted the number of training

points to 6000 as a compromise between accuracy and computational demand. Especially for PCA sampled training sets we observed only a small advantage of larger training sets measured by the validation set error. Several activation functions were probed in the Bayesian optimization.

1. *Convergence of Bayesian optimization runs*

Bayesian optimization selects a particular set of hyperparameters from all the possible values for each of the hyperparameters (see Tab. III). The MLP corresponding to this set of hyperparameters is then constructed and trained on the training set. Prediction errors on the validation set are used to evaluate the prediction accuracy of each constructed MLP after it was trained.

The Bayesian optimization procedure was run for a total time period of seven days (walltime) on four GPUs (NVIDIA Tesla K80) for each dataset (FMO, RC, CP43, CP47) and each training set selection method (random, PCA). We extract the validation set error for all MLPs generated and trained in this process. During optimization, we keep track of validation error of the current optimal MLP architecture. Fig. 2 to illustrate the progress of the hyperparameter optimization. After only a few iterations the prediction error for the validation set already has significantly dropped.

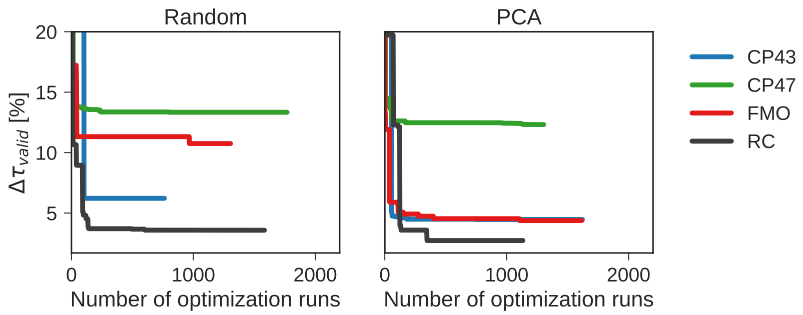

FIG. 2. Smallest relative validation error for the set of MLPs trained during the Bayesian Optimization over the number of MLP evaluations in the optimization.

In each of the Bayesian optimization procedures we did not see any decrease in the validation set errors for at least the last 200 proposed MLP architectures. Further, we observe that as opposed to a single best set of hyperparameters the Bayesian optimization instead reveals a number of MLP architectures with different hyperparameter values but similarly small validation set errors. Therefore, we conclude that the Bayesian optimization converged for all datasets and identified reasonably accurate MLP architectures.

2. *Bayesian optimization results*

We recorded the minimum validation set errors of all MLPs constructed and trained during the Bayesian optimization procedure to study the effect of particular hyperparameter choices on the prediction accuracy (see Fig. 3).

Optimal sets of hyperparameters resulting in the smallest validation set error for all four datasets with MLPs trained on PCA select (randomly drawn) training sets are reported in Tab. IV (Tab. V). While MLPs trained on the RC and the FMO datasets, which consists of fewer excitonic sites, tend to prefer shallow but broad architectures we achieved the smallest relative validation set errors with more hidden layers and fewer neurons per hidden layer for the CP43 and the CP47 dataset with more excitonic sites.

G. Comments on order ambiguities in Frenkel exciton Hamiltonians

As there is no well-defined ordering of excitonic sites in the considered system, Frenkel exciton Hamiltonians are only uniquely defined up to mutual permutations of rows and columns. This order ambiguity was found to be

Hyperparameter	RC	FMO	CP43	CP47
Training points	6000	6000	5480	6000
Layers	3	5	3	3
Neurons per layer	1729	1258	1899	749
Learning rate	$10^{-3.83}$	$10^{-3.44}$	$10^{-3.24}$	$10^{-3.19}$
Activation	softsign	relu	tanh	softsign
Regularization	$10^{-14.37}$	$10^{-18.0}$	$10^{-7.80}$	$10^{-2.91}$

TABLE IV. Optimized multi-layer perceptron (MLP) hyperparameters for the four investigated datasets (PCA selected training sets) used in the main text. Hyperparameters were optimized in a Bayesian optimization procedure

Hyperparameter	RC	FMO	CP43	CP47
Training points	6000	6000	6000	6000
Layers	3	3	3	7
Neurons per layer	901	1382	1913	962
Learning rate	$10^{-2.84}$	$10^{-3.21}$	$10^{-4.23}$	$10^{-4.08}$
Activation	softsign	softsign	softsign	softsign
Regularization	$10^{-18.0}$	$10^{-16.1}$	$10^{-12.0}$	$10^{-4.3}$

TABLE V. Optimized multi-layer perceptron (MLP) hyperparameters for the four investigated datasets (randomly selected training sets). Hyperparameters were optimized in a Bayesian optimization procedure

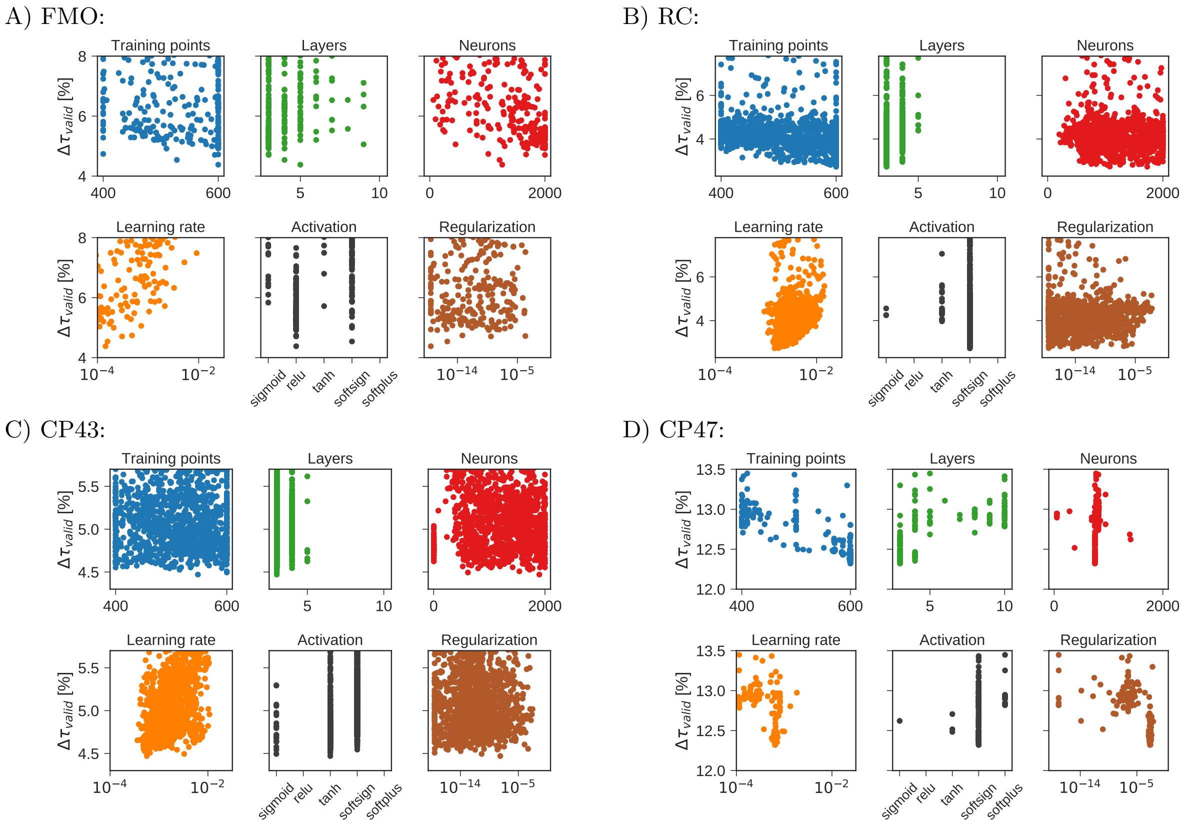

FIG. 3. Scatter plot of the average absolute relative errors of multi-layer perceptrons (MLPs) constructed during the Bayesian optimization procedure and trained on the indicated data set in dependence of the particular choices of hyperparameters made by the Bayesian optimizer. Each point represents the hyperparameters for the best architecture for each optimization step. MLPs were trained on PCA selected training sets.

challenging for MLPs trained to predict atomization energies from Coulomb matrices and different techniques have been developed to overcome this problem.[21].

Dataset	Permutation	$\Delta\tau_{\text{train}}$ [%]	$\Delta\tau_{\text{valid}}$ [%]	$\Delta\tau_{\text{test}}$ [%]
FMO	$\pi(1,8)$	182.1	178.4	186.9
	$\pi(3,8)$	180.7	169.4	172.5
	$\pi(6,8)$	8.1	7.9	7.6
	$\pi(7,8)$	8.0	8.0	8.2
	$\pi(6,7)$	8.0	7.9	7.8
RC	$\pi(1,8)$	32.5	31.7	33.1
	$\pi(3,8)$	32.2	31.5	31.8
	$\pi(6,8)$	3.2	3.1	3.4
	$\pi(7,8)$	3.2	3.2	3.4
	$\pi(6,7)$	3.2	3.1	3.4
CP43	$\pi(1,8)$	43.6	43.8	42.7
	$\pi(3,8)$	42.9	44.3	44.9
	$\pi(6,8)$	4.5	4.5	4.8
	$\pi(7,8)$	4.5	4.5	4.8
	$\pi(6,7)$	4.5	4.5	4.7
CP47	$\pi(1,8)$	111.9	121.5	114.8
	$\pi(3,8)$	116.9	123.5	114.4
	$\pi(6,8)$	12.4	12.5	12.7
	$\pi(7,8)$	12.4	12.5	12.6
	$\pi(6,7)$	12.4	12.5	12.5

TABLE VI. Average relative absolute errors ($\Delta\tau$) of exciton transfer times computed with HEOM and predicted by MLPs trained on PCA selected training sets. Before passing each dataset to the MLPs for prediction we performed permutations $\pi(i, j)$ on each Hamiltonian by interchanging the i-th and j-th rows and columns. Note, that site 1 was set to be the initial site for population dynamics and site 3 was the target site.

In this study, Frenkel exciton Hamiltonians were constructed by uniformly sampling excited state energies and inter-site couplings. For all sites in a system we used the same sampling distributions. In the case of Coulomb matrices, however, the distributions of values for diagonal and off-diagonal elements can be different for different atom types, which is why the ordering ambiguity emerges.

To illustrate that the ordering ambiguity does not arise for the Frenkel exciton Hamiltonian, we used our trained MLPs to predict exciton transfer times and efficiencies for all exciton Hamiltonians in each dataset for which we permuted some of the rows and columns. Note, that we chose site 1 as the initially excited site for the population dynamics simulations while site 3 acted as an acceptor. Permutations involving sites 1 or 3 are therefore expected to significantly change the prediction results. The obtained average relative absolute prediction errors are reported in Tab. VI.

We generally observe prediction errors for permuted Hamiltonians comparable to the prediction errors on the unpermuted test sets if the permutations are performed on sites other than 1 and 3. If, however, sites 1 or 3 were involved in the permutation the prediction errors drastically increased by at least an order of magnutide.

H. Comparison of exciton transfer times obtained from exciton dynamics calculations and multi-layer perceptron predictions

We trained MLP models to predict exciton transfer times and efficiencies from Frenkel exciton Hamiltonians to provide an alternative to computationally costly exciton dynamics calculations. In this section, we comment on the prediction accuracy of MLP models by comparing their predictions with results obtained from two popular exciton dynamics approaches, the secular Redfield method and the hierarchical equations of motion (HEOM) formalism (on which the MLPs were trained). While secular Redfield is known to underestimate exciton transfer times it is computationally cheaper than HEOM.

We provide a context for MLP prediction accuracies by comparing MLP predicted transfer times to transfer times obtained from secular Redfield and HEOM calculations on the level of individual Hamiltonians. Fig. 4 shows scatter plots of transfer times obtained from all three approaches. We plot transfer times predicted by MLPs and secular Redfield versus transfer times calculated with HEOM, which we consider as the ground truth for the purpose of this study. The green line in Fig. 4 indicates perfect agreement between HEOM predictions and predictions by either

MLPs or secular Redfield.

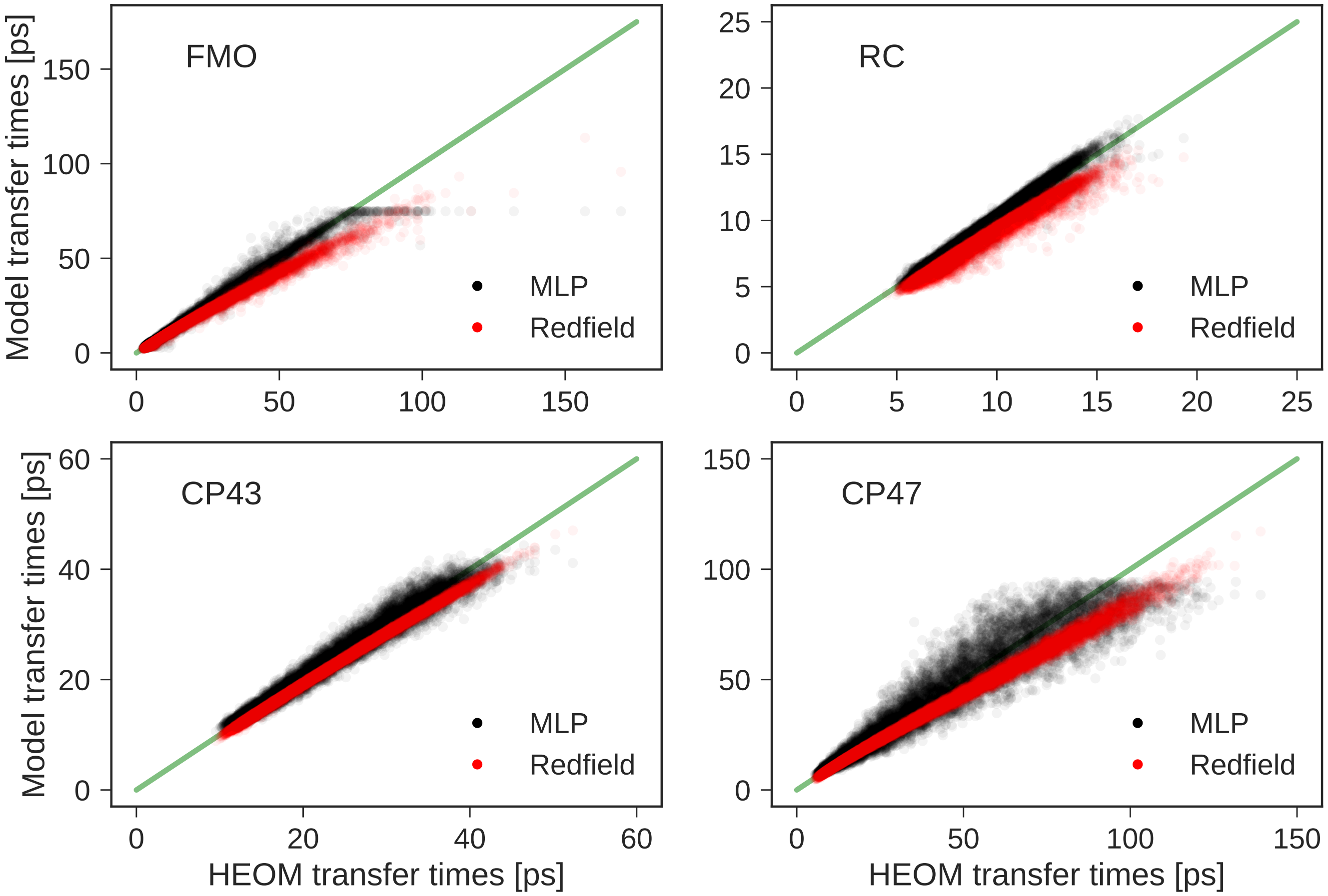

FIG. 4. Exciton transfer times as computed with the hierarchical equations of motion (HEOM) approach compared to ecxiton transfer times calculated with the secular Redfield method or predicted from trained multi-layer perceptrons (MLPs). Panels show the exciton transfer times obtained for all 12000 exciton Hamiltonians in each of the four generated datasets. The griin line indicates perfect agreement between HEOM results and predictions by either MLPs or secular Redfield.

By comparing the exciton transfer time predictions of the secular Redfield approach to the exciton transfer times of HEOM we observe that secular Redfield almost always underestimates transfer times consistently in all four datasets. MLPs instead over- and underestimate exciton transfer times, which is due to the symmetric loss function employed during MLP training and hyperparameter optimization.

The absolute deviation between Redfield and HEOM transfer times generally increases with the value of the exciton transfer time, as observed in previous studies.[16] Also for the MLP predictions, we observe a larger deviation from the HEOM results for longer transfer times. However, in contrast to secular Redfield, the error is still distributed rather symmetrically around our ground truth. This observation is explained by the fact that MLPs were trained to minimize the relative deviation between predicted and HEOM transfer times. That is, for longer transfer times the predictions can afford larger deviations from HEOM which results in an opening funnel structure, especially seen in the scatter plot for the CP47 dataset. In addition, all of the presented datasets include fewer data points at larger transfer times, but MLPs are trained to minimize loss functions which take the unweighted average over all transfer times.

[1] V. May and O. Kühn. *Charge and Energy Transfer Dynamics in Molecular Systems*. Wiley-VCH, Weinheim, 2004.

[2] Y. C. Cheng and G. R. Fleming. Dynamics of light harvesting in photosynthesis. *Annu. Rev. Phys. Chem.*, 60:241–262, 2009.
[3] J. A. Leegwater. Coherent versus incoherent energy transfer and trapping in photosynthetic antenna complexes. *J. Phys. Chem.*, 100:14403–14409, 1996.
[4] V. May and O. Kühn. *Charge and energy transfer dynamics in molecular systems*. John Wiley & Sons, 2008.
[5] Y. Yan, F. Yang, Y. Liu, and J. Shao. Hierarchical approach based on stochastic decoupling to dissipative systems. *Chem. Phys. Lett.*, 395:216–221, 2004.
[6] R. Xu, P. Cui, C. Li, Y. Mo, and Y. Yan. Exact quantum master equation via the calculus on path integrals. *J. Chem. Phys.*, 112:041103, 2005.
[7] A. Ishizaki and Y. Tanimura. Quantum dynamics of system strongly coupled to low-temperature colored noise bath: Reduced hierarchy equations approach. *J. Phys. Soc. Jap.*, 74:3131–3134, 2005.
[8] A. Ishizaki and G. R. Fleming. Unified treatment of quantum coherent and incoherent hopping dynamics in electronic energy transfer: Reduced hierarchy equation approach. *J. Chem. Phys.*, 130:234111, 2009.
[9] C. Kreisbeck, T. Kramer, M. Rodriguez, and B. Hein. High-performance solution of hierarchical equations of motion for studying energy transfer in light-harvesting complexes. *J. Chem. Theory Comput.*, 7:2166–2174, 2011.
[10] C. Kreisbeck and T. Kramer. Long-lived electronic coherence in dissipative exciton dynamics of light-harvesting complexes. *Phys. Chem. Lett.*, 3:2828–2833, 2012.
[11] C. Kreisbeck, T. Kramer, and A. Aspuru-Guzik. Scalable high-performance algorithm for the simulation of exciton dynamics. application to the light-harvesting complex ii in the presence of resonant vibrational modes. *J. Chem. Theory Comput.*, 10:4045–4054, 2014.
[12] C. Kreisbeck and A. Aspuru-Guzik. Efficiency of energy funneling in the photosystem ii supercomplex of higher plants. *Chem. Sci.*, 7:4174–4183, 2016.
[13] S. M. Blau, D. I. G. Bennett, C. Kreisbeck, G. D. Scholes, and A. Aspuru-Guzik. Local protein solvation drives direct down-conversion in phycobiliprotein pc645 via incoherent vibronic transport. *arXiv:1704.05449*, 2017.
[14] C. Kreisbeck, T. Kramer, and A. Aspuru-Guzik. Disentangling electronic and vibronic coherences in two-dimensional echo spectra. *J. Phys. Chem. B*, 117:9380–9385, 2013.
[15] H.-P. Breuer and F. Petruccione. *The theory of open quantum systems*. Oxford University Press on Demand, 2002.
[16] V. Novoderezhkin, A. Marin, and R. van Grondelle. Intra- and inter-monomeric transfers in the light harvesting lhcii complex: the redfield-förster picture. *Phys. Chem. Chem. Phys.*, 13:17093–17103, 2011.
[17] J. Adolphs and T. Renger. How proteins trigger excitation energy transfer in the FMO complex of green sulfur bacteria. *Biophys. J.*, 91:2778–2797, 2006.
[18] F. Müh, M.E.A. Madjet, and T. Renger. Structure-based simulation of linear optical spectra of the cp43 core antenna of photosystem ii. *Photosynth. Res.*, 111(1-2):87–101, 2012.
[19] G. Raszewski and T. Renger. Light harvesting in photosystem ii core complexes is limited by the transfer to the trap: can the core complex turn into a photoprotective mode? *J. Am. Chem. Soc.*, 130(13):4431–4446, 2008.
[20] G. Raszewski, B. A. Diner, E. Schlodder, and T. Renger. Spectroscopic properties of reaction center pigments in photosystem ii core complexes: revision of the multimer model. *Biophys. J.*, 95(1):105–119, 2008.
[21] K. Hansen, G. Montavon, F. Biegler, S. Fazli, M. Rupp, M. Scheffler, O. A. von Lilienfeld, A. Tkatchenko, and K. R. Müller. Assessment and validation of machine learning methods for predicting molecular atomization energies. *J. Chem. Theory Comput.*, 9:3404—3419, 2013.
